# A Machine Learning Aided Systematic Review and Meta-Analysis of the Relative Risk of Atrial Fibrillation in Patients With Diabetes Mellitus

**DOI:** 10.3389/fphys.2018.00835

**Published:** 2018-07-03

**Authors:** Zhaohan Xiong, Tong Liu, Gary Tse, Mengqi Gong, Patrick A. Gladding, Bruce H. Smaill, Martin K. Stiles, Anne M. Gillis, Jichao Zhao

**Affiliations:** ^1^Auckland Bioengineering Institute, The University of Auckland, Auckland, New Zealand; ^2^Department of Cardiology, Second Hospital of Tianjin Medical University, Tianjin, China; ^3^Key Laboratory of Ionic-Molecular Function of Cardiovascular Disease, Tianjin Institute of Cardiology, Tianjin, China; ^4^Department of Medicine and Therapeutics, Li Ka Shing Institute of Health Sciences, The Chinese University of Hong Kong, Shatin, Hong Kong; ^5^Department of Cardiology, Waitemata District Health Board, Auckland, New Zealand; ^6^Waikato Hospital, Hamilton, New Zealand; ^7^Department of Cardiac Sciences, Libin Cardiovascular Institute of Alberta, University of Calgary, Calgary, AB, Canada

**Keywords:** atrial fibrillation, diabetes mellitus, meta-analysis, machine learning, risk factor

## Abstract

**Background:** Meta-analysis is a widely used tool in which weighted information from multiple similar studies is aggregated to increase statistical power. However, the exponential growth of publications in key areas of medical science has rendered manual identification of relevant studies increasingly time-consuming. The aim of this work was to develop a machine learning technique capable of robust automatic study selection for meta-analysis. We have validated this approach with an up-to-date meta-analysis to investigate the association between diabetes mellitus (DM) and new-onset atrial fibrillation (AF).

**Methods:** The PubMed online database was searched from 1960 to September 2017 where 4,177 publications that mentioned both DM and AF were identified. Relevant studies were selected as follows. First, publications were clustered based on common text features using an unsupervised K-means algorithm. Clusters that best matched the selected set of potentially relevant studies (a “training” set of 139 articles) were then identified by using maximum entropy classification. The 139 articles selected automatically on this basis were screened manually to identify potentially relevant studies. To determine the validity of the automated process, a parallel set of studies was also assembled by manually screening all initially searched publications. Finally, detailed manual selection was performed on the full texts of the studies in both sets using standard criteria. Quality assessment, meta-regression random-effects models, sensitivity analysis and publication bias assessment were then conducted.

**Results:** Machine learning-assisted screening identified the same 29 studies for meta-analysis as those identified by using manual screening alone. Machine learning enabled more robust and efficient study selection, reducing the number of studies needed for manual screening from 4,177 to 556 articles. A pooled analysis using the most conservative estimates indicated that patients with DM had ~49% greater risk of developing AF compared with individuals without DM. After adjusting for three additional risk factors i.e., hypertension, obesity and heart disease, the relative risk was 23%. Using multivariate adjusted models, the risk for developing AF in patients with DM was similar for all DM subtypes. Women with DM were 24% more likely to develop AF than men with DM. The risk for new-onset AF in patients with DM has also increased over the years.

**Conclusions:** We have developed a novel machine learning method to identify publications suitable for inclusion in meta-analysis.This approach has the capacity to provide for a more efficient and more objective study selection process for future such studies. We have used it to demonstrate that DM is a strong, independent risk factor for AF, particularly for women.

## Introduction

Meta-analysis is a powerful epidemiological tool that is increasingly used in all fields of scientific research. It seeks to amplify statistical power by aggregating weighted information from multiple similar studies (Moher et al., 2009). This approach is driven by the view that common trends, masked by potential error in individual scientific investigations, will be revealed if sufficient numbers of conceptually similar studies are combined and different sources of error are appropriately accounted for. The exponential growth of publications in key areas of medical science offers important new opportunities to extend the scope of meta-analyses, however, it has also rendered conventional manual identification of relevant studies increasingly time-consuming. Furthermore, the selection of studies for meta-analysis must be based on objective criteria and bias that is introduced from manual selection can affect results.

Atrial fibrillation (AF) is the most commonly sustained arrhythmia that is associated with substantial morbidity and mortality (Colilla et al., 2013). In the developed world, one in five strokes in people aged over 60 years is associated with AF (Colilla et al., 2013; Krijthe et al., 2013). The overall prevalence of AF is currently ~2% of the general population worldwide and is projected to more than double in the next four decades due to the aging population and the increasing incidence of other concurrent diseases (Colilla et al., 2013; Krijthe et al., 2013). Risk factors of AF include age, hypertension, obesity, valvular heart disease, heart failure and obstructive sleep apnea (Movahed et al., 2005). The relationship between AF and diabetes mellitus (DM) is complex since both diseases are associated with confounders, such as hypertension, obesity and vascular disease (Schoen et al., 2012). Some (Krahn et al., 1995; Kannel et al., 1998; Movahed et al., 2005; Aksnes et al., 2008; Huxley et al., 2011) but not all epidemiological studies (Frost et al., 2005; Fontes et al., 2012; Huxley et al., 2012; Johnson et al., 2014) have suggested that DM represents an independent risk factor for AF. However, these studies have been limited by their diverse research designs and lack of a sufficient number of enrolled patients. Individually, no study has established overwhelming evidence of the association.

The causality of the relationship between DM and AF was initially investigated by a small-scale meta-analysis (Huxley et al., 2011) that included only six prospective cohort studies and four case–control studies with significant heterogeneity selected from 482 publications searched in 2010. Over the past 8 years, more than 2,000 additional publications have reported the association between DM and AF. These additional data samples enhance the statistical power of meta-analyses to determine the association between DM and AF, but they also amplify the burden of selection at the same time. The aims of this study were therefore to develop a novel machine learning technique to provide a more efficient and robust study selection method for meta-analysis, and to perform an up-to-date meta-analysis to determine the association between DM and new-onset AF.

## Materials and methods

### Data sources and searches

We conducted this study in accordance with Preferred Reporting Items for Systematic Review and Meta-Analysis (PRISMA) guidelines (Moher et al., 2009) (refer to the PRISMA 2009 Flow Diagram in Figure S1). A strategic search was conducted on the PubMed online database of publications from 1960 to 1 September 2017. The search included any studies that contained the keywords “diabetes”/“diabetic” and “atrial fibrillation” in any field without language restriction. This identified a wide range of research studies that involved both DM and AF.

### Study selection criteria

Study selection was limited to cohort studies, randomized trials and case-control studies in adult populations (participants aged >18 years old) where an association between DM and AF was reported. Patients with established comorbidities such as cardiac disease, hypertension and obesity/body mass index (BMI) were included so that the potential influence of these conditions on AF could be evaluated and the adjusted risk ratios were calculated. To reduce publication bias, previous review and meta-analysis papers were excluded. Furthermore, where multiple findings were reported from the same or overlapping patient datasets, the most contemporary study was used.

### Machine learning assisted study selection and validation

**Figure 1:**
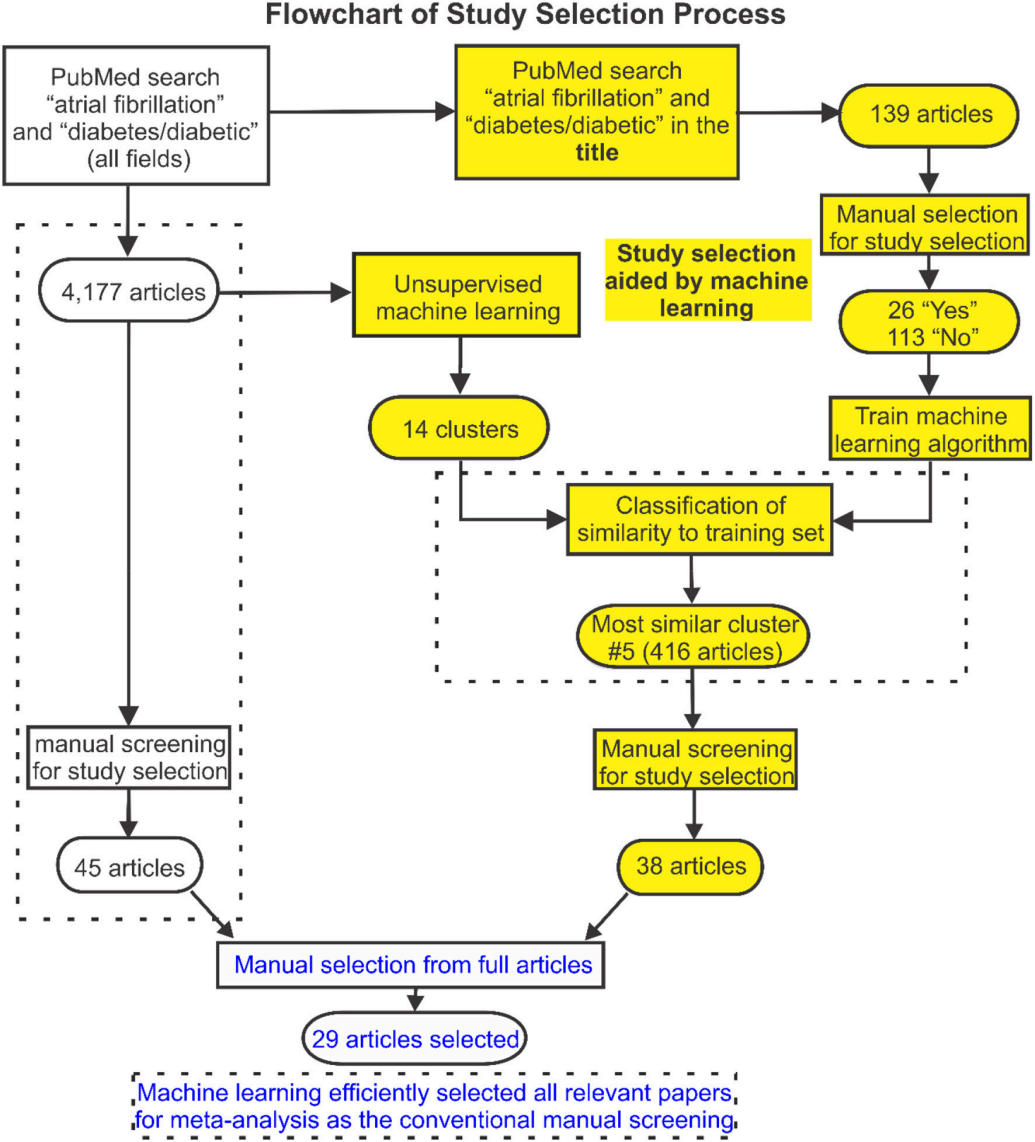
Flowchart of search and selection strategy used in this study. The study selection flowchart is displayed here, in which a machine learning approach was developed to facilitate the publication selection. Articles from searched publications were first grouped into 14 clusters by unsupervised machine learning. Then supervised machine learning was used to identify clusters of articles with greatest relevance to the labeled training set identified based on a subset of articles from the initial search. Full texts of the studies identified were then reviewed and 29 articles were selected for the meta-analysis which was validated by the conventional manual selection approach.

**Figure 2:**
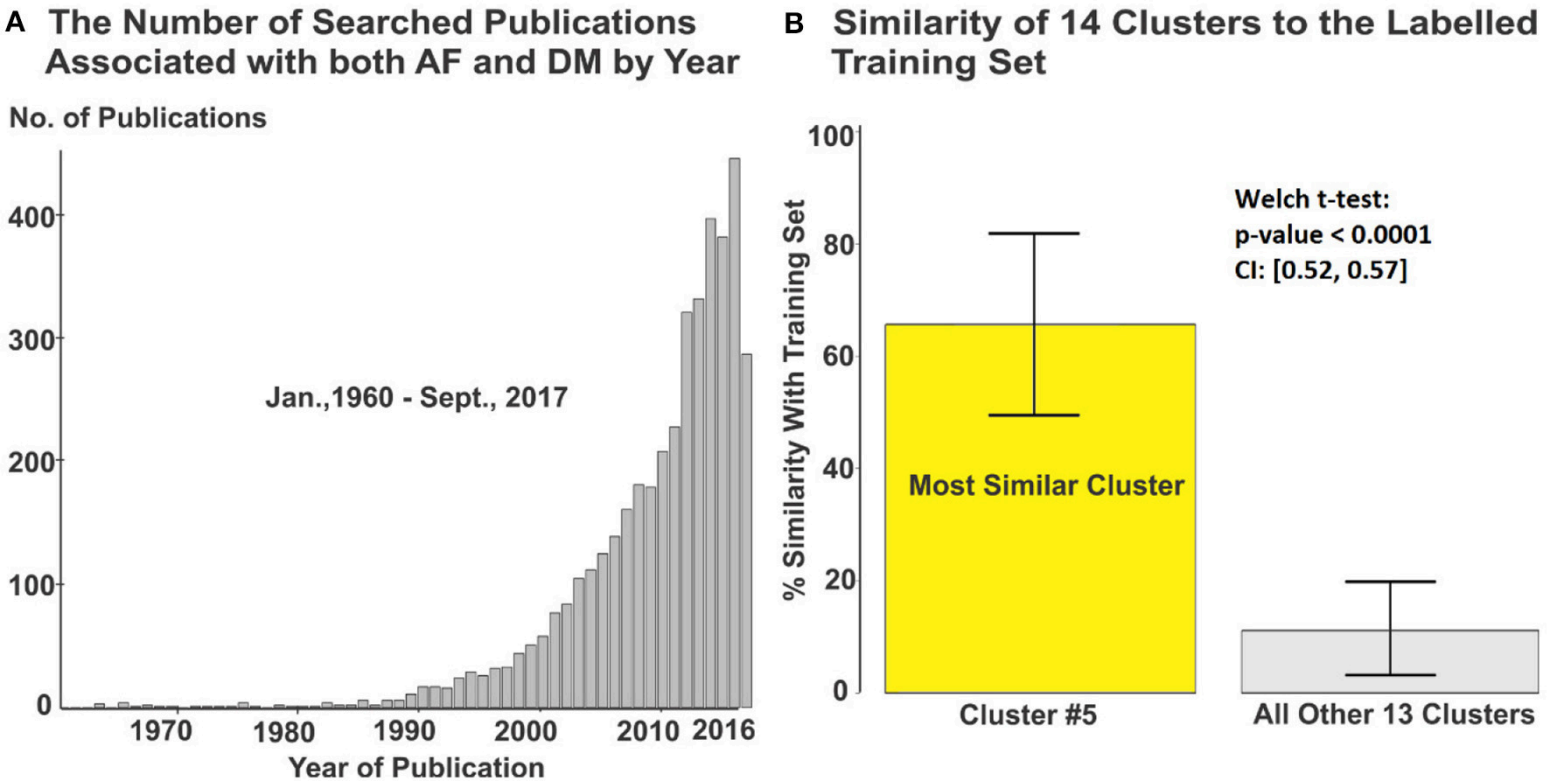
**(A)** 4,177 articles from originally searched publications were used in the meta-analysis. **(B)** Cluster #5 containing 416 articles was automatically identified with greatest relevance to the labeled training set. DM, diabetes milieus; AF, atrial fibrillation.

Conventionally, study selection is a two-stage process. During the first stage, titles and abstracts of all articles returned from the initial strategic search are manually screened to decide whether they potentially meet the study selection criteria. Full texts of the subset of studies identified are then reviewed in detail and those that meet the criteria are selected. Decisions are made by two experts at both stages. Any conflict was referred to a third expert and resolved by discussion and consensus. A novel machine learning approach was developed to automate the first-stage screening process and to facilitate the process of study selection. This was implemented in *R*, an open-source environment for data mining, text processing, machine learning and statistical analysis (R Core Team, 2013). The approach used is outlined schematically in Figures 1, 2, where a more detailed description is provided in the Supplementary Methods.

Articles returned by the PubMed search were grouped into clusters with common attributes (Figure 1). A well-established feature detection method was used to assign weighting factors based on the frequency of occurrence of words and word combinations in the PubMed texts (including titles and abstracts). An unsupervised K-means clustering algorithm was utilized to group publications with similar content (based on these weightings) and sort them into a limited number of clusters. This was done by iteratively removing the smallest cluster of publications and re-clustering the remainder until the number of remaining publications was < 250. The residual studies were then grouped as a remainder cluster.

A subset of articles from the initial search was extracted as a training set for machine learning-assisted screening. First, articles most likely to be relevant were identified by searching for the keywords “diabetes”/“diabetic” and “atrial fibrillation” in the title. Titles and abstracts of these articles were then screened manually and labeled as potentially meeting or not meeting the selection criteria. Supervised machine learning (maximum entropy classification) was used to fit a predictive model to the labeled training set and this was applied across all clusters to identify clusters of articles that best matched this selection (Figure 2). Titles and abstracts of the articles in these clusters were then screened manually to select relevant studies for further study. Finally, full texts of the studies identified were reviewed and selections were made according to the stated criteria, removing any duplicate studies in the process.

To validate our machine learning assisted screening approach, study selection using this approach was compared to the results of conventional manual selection (see Figure 1). Identical manual screening and selection procedures were used in both study arms. The Newcastle-Ottawa or modified Jadad Scale was used to evaluate the quality of the included studies.

### Data synthesis and analysis

Baseline demographics collected from individual studies included the nature of the study (cohort/case-control/randomized), year of subject's enrolment, country of the study, number of subjects, AF subtypes, DM subtypes, sex, age, year of follow up and comorbidities. Quantitative estimates of the association between DM and AF were also extracted from the original publications together with their respective confidence intervals (CIs). These included either hazard ratios (HR) for cohort/randomized studies or odds ratio (OR) for retrospective case-control studies. Age-and/or-gender/none adjusted (minimal adjusted) estimates were extracted from individual studies as well as other multivariate adjusted estimates when provided. Relative risk (RR) was estimated using the DerSimonian and Laird random-effects model across different studies and subgroups such as men vs. women.

The most conservative estimates provided in individual studies were used in the meta-analysis to take advantage of all included studies. Multivariate adjusted estimates were applied wherever possible using a subset of these studies by utilizing the studies with age, sex, and additional multiple adjustments for various reported risk factors. Furthermore, risk estimates for AF were adjusted separately for hypertension, obesity and various heart diseases in addition to other adjustments.

### Sensitivity analysis and publication bias

The effects on RR of CIs, publication year, year of the study, age, mean follow-up years and number of adjusted factors were investigated using polynomial regression analysis with Pearson's correlation. Throughout this study, statistical significance was assessed using the 2-tailed *T*-test. Publication bias was assessed using the Egger regression test and is presented as a funnel plot.

## Results

### Validation of machine learning-assisted study selection

The initial PubMed search yielded a total of 4,177 publications and these were automatically sorted into 14 groups using unsupervised clustering. The training dataset consisted of 139 articles of which 26 were labeled as potentially relevant and 113 not relevant (Figure 1). Using machine learning, it was found that one cluster (#5) had substantially greater similarity to the 26 studies identified as potentially relevant in the training set than all others (Figure 2B, Figure S2). Manual screening of the titles and abstracts of the 416 articles in this cluster resulted in 38 being selected as potentially relevant compared to the 45 selected following direct manual screening of all 4,177 articles. Manual review of the full articles in both cases yielded the same 29 final selections (Details of these studies are provided in Tables S1, S2).

The 29 articles selected for meta-analysis (Tables S1, S2) had a study population of 8,037,756. Further details of the 9 studies that were excluded following machine learning assisted screening are given in Table S3. Scores for the quality assessment (Newcastle-Ottawa quality assessment scale (NOS)*/*modified Jadad score) ranged from 5 to 9 for the cohort/randomized/case-control studies (9 representing the highest quality). The overall average score was 7.4 (Tables S4, S5).

### Risk estimates of new-onset AF in patients with DM

#### Baseline estimates

**Figure 3:**
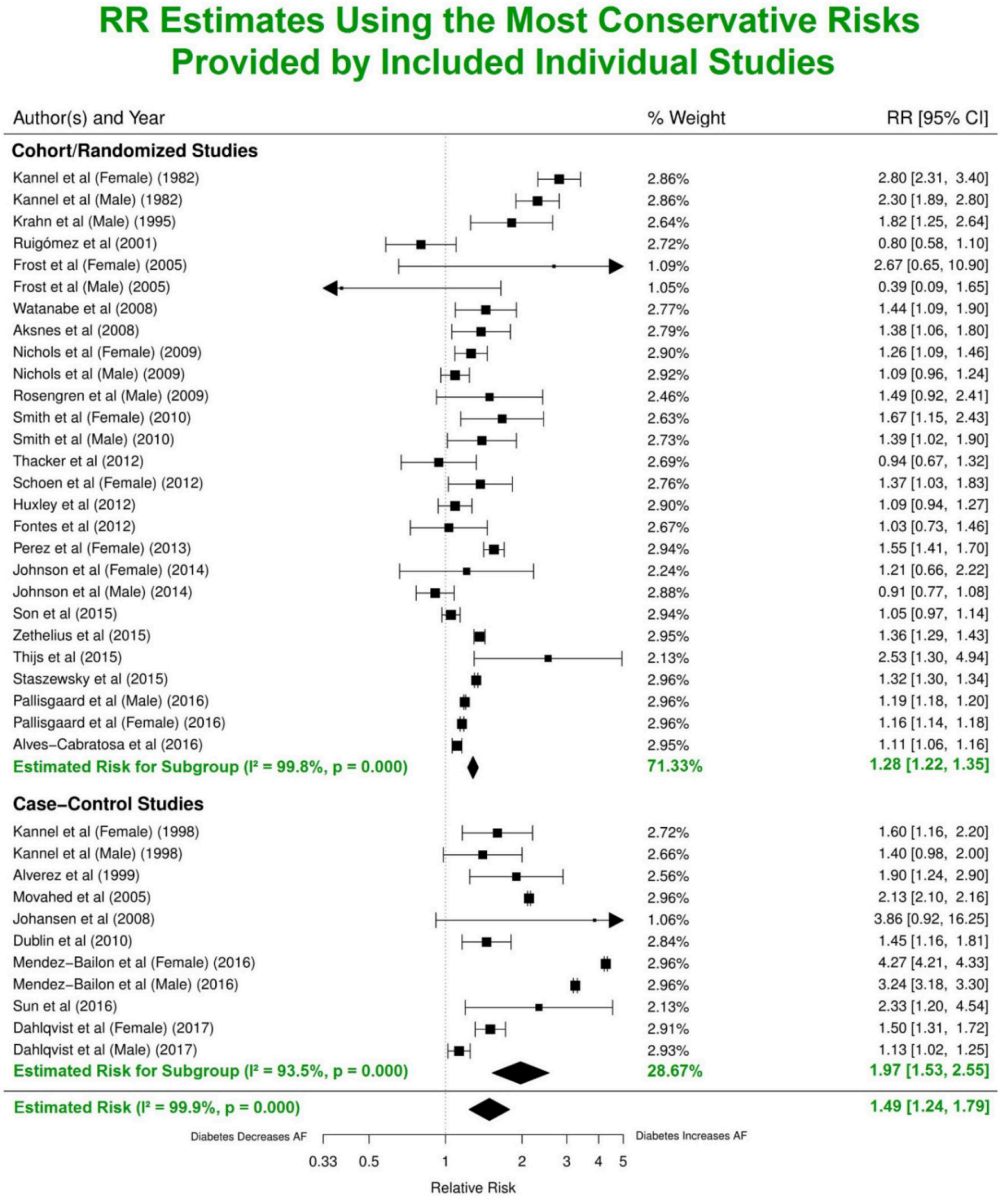
Estimated risks of AF in patients with DM using the most conservative risk estimates provided in the individual studies. Subgroup summaries for cohort/randomized and case-control studies are in bold at the bottom of each subgroup. DM, diabetes milieus; AF, atrial fibrillation; RR, relative risk; CI, confidence interval.

Analysis of the combined cohort, randomized and case-control studies identified that patients with DM had ~49% greater risk of developing AF (RR 1.49, 95% CI 1.24–1.79) compared to individuals without DM (Figure 3). The forest plot shows a significantly lower risk in the cohort/randomized studies (RR 1.28, 95% CI 1.22–1.35) compared to the *eight* case control studies (OR 1.97, 95% CI 1.53–2.55, *p* < 0.01). Funnel plot assessment (Figure S3A) provided evidence for no publication bias (*p* = 0.87, *z* = 0.16).

#### Estimates adjusted for confounding risks

Nine studies were adjusted for risk estimates based only on age-and/or-gender/none, while 20 included adjustments for multiple risk factors including different combinations of hypertension, BMI, height, smoking, blood pressure, alcohol consumption, various cardiac diseases and race (Figure S4). The levels of adjustment used in different publications influenced the calculated RRs. For studies with minimal adjustments (only age-and/or-gender/none), the summary estimate of risk of AF was significantly higher (RR 2.28, 95% CI 1.95–2.67), compared with studies that included adjustments for additional risk factors (RR 1.25, 95% CI 1.12–1.41).

**Figure 4:**
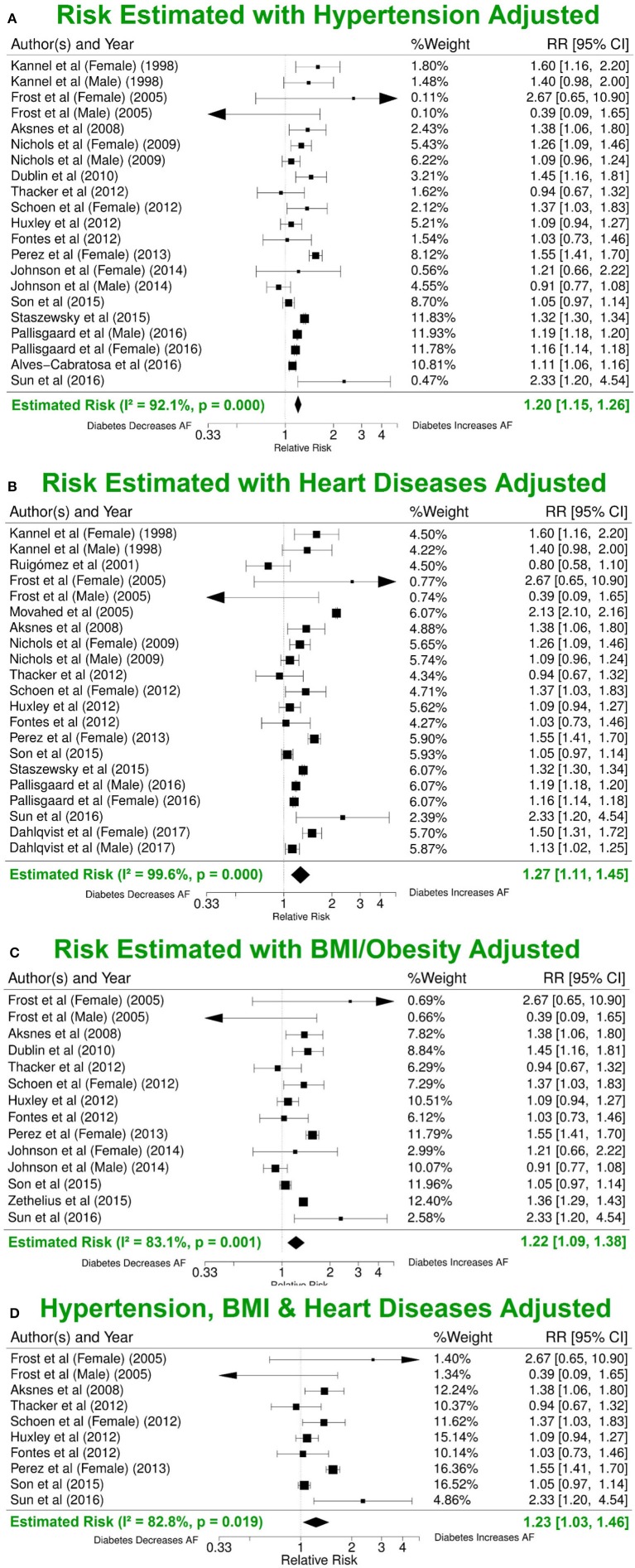
Risk estimates with different additional risk factor adjustments. **(A)** Forest plot of risk values adjusted for hypertension in addition to age-and/or-sex/none and other included risk factors. **(B)** RR estimate adjusted for BMI in addition to age-and/or-sex/none and other included risk factors. **(C)** RR estimate adjusted for various heart conditions in addition to age-and/or-sex/none and other included risk factors. **(D)** Summary estimate for RRs after adjusting for hypertension, BMI and various heart conditions in addition to age-and/or-sex/none and other included risk factors. BMI, body mass index; DM, diabetes milieus; AF, atrial fibrillation; RR, relative risk; CI, confidence interval.

Hypertension, cardiac disease, and obesity are known risk factors for AF (Movahed et al., 2005). Analysis of the studies that reported risk of AF after adjusting for at least one of the three common risk factors yielded lower RRs (1.20, 95% CI 1.15–1.26; 1.27, 95% CI 1.11–1.45; 1.22, 95% CI 1.09–1.38) (Figures 4A–C). Nine publications with reduced study heterogeneity of 82.8% (*I*^2^ statistic) included adjustments for all the three factors and the estimated overall risk of AF in patients with DM was lower (RR 1.23, 95% CI 1.03-1.46) (Figure 4D).

### Impact of study demographics on AF incidence

#### Impact of major demographical components

The effects on RR of the width of the CIs (number of enrolled patients), publication year, study location, year of patient enrollment, AF subtype, DM subtype, sex, age and mean follow-up year were also investigated. Continental location (Figure S3B) had no significant impact on RRs (*p* = 0.8), and mean follow-up duration displayed a minor impact (Figure S3C). Furthermore, we observed an inverse relationship between the number of adjusted risk factors and estimated RRs, as well as between the CI widths and RRs. Removing the study with the largest population (Pallisgaard et al., 2016) (narrowest CI) or the paper with relative higher/lower risk estimate led to negligible changes in the overall risk estimates. Polynomial regression analysis yielded a positive linear correlation between age and the RR (Pearson's correlation: *R*^2^ = 0.32, *p* = 0.049).

#### Impact of AF and DM subtypes

**Figure 5:**
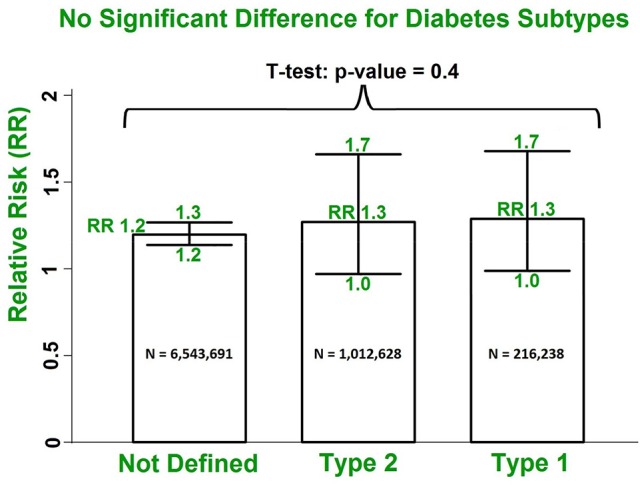
No significant difference in risks of AF incidence in patients with DM for undefined DM subtypes, type 2 DM and type 1 DM using the multivariate model. DM, diabetes milieus; AF, atrial fibrillation; RR, relative risk.

There were only 4 studies exploring the linkage between DM and a specific AF subtype, compared with 27 studies reporting the relationship between DM and AF (all subtypes). Our results with multivariate adjustment for confounders found no significant difference (*p* = 0.5) between the risks of the different AF subtypes (RR 1.4, 95% CI 1.0–1.8; RR 1.3, 95% CI 1.0–1.8; RR 1.3, 95% CI 1.0–1.9 for paroxysmal/persistent/permanent AF, respectively) in patients with DM, compared with all subtypes of AF (RR 1.3, 95% CI 1.1–1.5). Similarly, using the multivariate risk model, no significant difference in the AF risk estimate (*p* = 0.4) was observed in the studies grouped by undefined DM subtypes (*N* = 6,543,691), DM type 2 (*N* = 1,012,628), and DM type 1 (*N* = 216,238) (Dahlqvist et al., 2017) (Figure 5). The estimated RRs were 1.2 (95% CI 1.2–1.3), 1.3 (95% CI 1.0–1.7) and 1.3 (95% CI 1.0–1.7) for the three subgroups, respectively (Figures S5, S6).

#### Impact of gender and enrolment date

**Figure 6:**
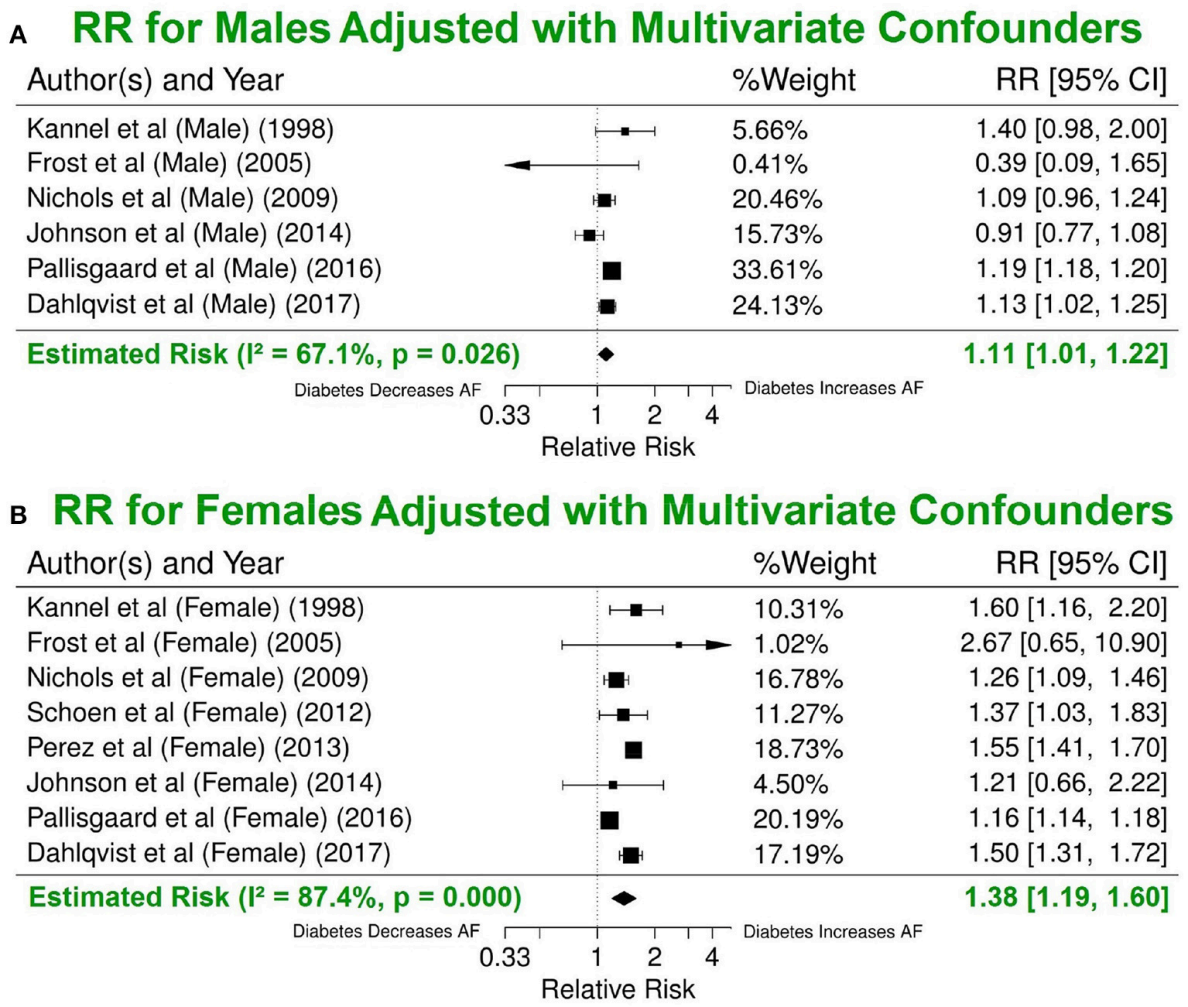
Significant difference in the risk of AF incidence between men and women with DM using the multivariate model. Summary estimate for publications that reported risk values for men **(A)** and for women **(B)**. DM, diabetes milieus; AF, atrial fibrillation; RR, relative risk; CI, confidence interval.

**Figure 7:**
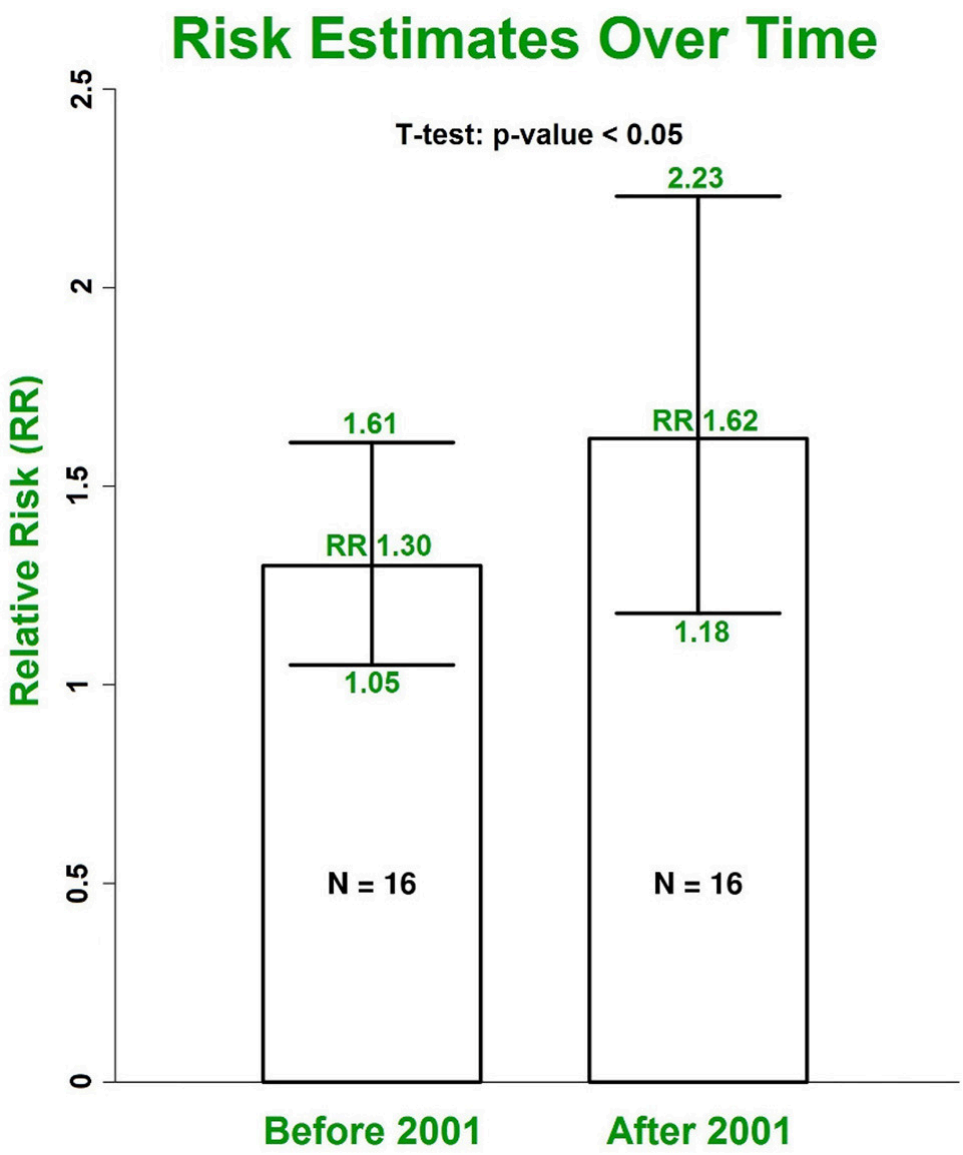
The increasing trend for RRs of AF in patients with DM grouped by the median year of patient enrolment. The risk was estimated using the most conservative risks provided in included individual studies. DM, diabetes milieus; AF, atrial fibrillation; RR, relative risk.

Our results with multivariate adjustment for confounders show a higher risk of AF in women (RR 1.38, 95% CI 1.19–1.60) compared to men (RR 1.11, 95% CI 1.01–1.22, *p* < 0.001) (Figure 6). Analysis of the median year of patient enrolment in the 23 studies from the past 35 years identified an increasing risk of AF over time in patients with DM using the most conservative adjustment (Figure 7). The RR estimated for the most recent studies (2001–2016) was significantly higher than for the studies prior to 2001 (RR 1.62, 95% CI 1.18–2.23 vs. RR 1.30 95%, CI 1.05–1.61, *p* < 0.05).

## Discussion

This systematic review and meta-analysis systematically analyzed 29 studies with a total of 8,037,756 participants from three continents, selected from 4,177 articles returned from an initial strategic search. To our knowledge, this is the largest study of this kind to explore the association between AF and DM, and the first to employ a machine learning approach to facilitate study selection.

### Benefits and validation of machine learning approach for study selection

From our literature research, the total number of publications related to the association investigated here has increased more than 4-fold over the last 10 years. The growth in medical research publications is accelerating across the board and we expect that it will no longer be feasible in the near future to maintain current manual study selection methods for large-scale meta-analyses and systematic review. Therefore, there is an urgent need to develop an intelligent automated approach, such as machine learning, to facilitate identification and selection of relevant articles for this purpose.

In this study, we have used machine learning to assist the screening of potentially relevant articles for large-scale meta-analyses and systematic review. To the best of our knowledge, we are the first to employ such an approach to facilitate study selection. With our approach, the burden of manual screening is reduced from all the articles returned by the initial online strategic search to those in the training set and in the principal cluster(s) identified by supervised machine learning. In this study, the number of publications for which manual screening was needed reduced from 4,177 to 555 using machine learning assisted screening, i.e., an 87% reduction. The iterative clustering approach (unsupervised machine learning) utilized vastly improved the similarity of the articles in individual clusters. Subsequent use of supervised machine learning (maximum entropy classification) with a representative training data subset enabled us to screen articles more rapidly than conventional manual procedures.

Automated methods of meta-analyses have been proposed in previous studies; however, few have automated the most labour intensive initial study screening stage. A study by Michelson (2014) proposed a method in which numerical features from different articles were first extracted into a matrix, and the matrices from of different articles were then grouped using a clustering algorithm to sort studies with similar content into the same group. The initial features were extracted by creating a program which carefully defined sets of rules based on patterns in the wording of the articles. Although this study also used an automated method, it differs significantly from our study. Manually setting decision criteria for extracting specific features about each article is time consuming, and does not generalize well to new data with different formats; while on the other hand, our proposed methods extracted generalized features not dependent on any individual study. Previous studies have also proposed automated methods of text mining to aid the initial screening stage of meta-analyses (Ananiadou et al., 2009; Kim et al., 2011; Thomas et al., 2011; Cohen et al., 2012; García Adeva et al., 2013) as we did in our systematic review. However, for their proposed supervised machine learning, a manually selected initial training set will still be required to automate the remaining literature search (Ananiadou et al., 2009; Cohen et al., 2012; García Adeva et al., 2013). Furthermore, their studies were not validated in a large scale meta-analysis. Our study utilizes an ensemble of methodologies including text mining, clustering and supervised machine learning to efficiently extract the relevant literature, and was verified as the literature extracted from our algorithm was the same as the literature identified through manual search. Furthermore, our results were also validated by comparing the risk estimates produced from the meta-analysis which was consistent with prior clinical studies.

A further benefit of machine learning-assisted selection of studies for meta-analysis is that identical screening can be applied and consistent results will be obtained if the meta-analysis is recapitulated with additional articles, minimizing the introduction of time-related bias. Machine learning-assisted screening therefore has the capacity to provide more efficient and more objective study selection for future meta-analyses. We expect that the novel automated approaches demonstrated will be extended and refined in the future.

### Impact of comorbidities on risk estimate

AF is associated with several well-established risk factors including age, hypertension, BMI and various cardiovascular diseases. The RRs assessed for these established risk factors have varied between studies and over time. Recent data from the Framingham Heart Study indicate that mean systolic blood pressures and evidence of left ventricular hypertrophy have declined, likely as a consequence of improved therapy for hypertension. In contrast, increasing BMI and DM have contributed to increased risk of AF in the population (Schnabel et al., 2015; Lau et al., 2017).

For reliable estimation of the independent risk of AF due to DM, it is necessary to adjust for these established risk factors of AF. In our study, patients with DM have an overall adjusted RR of 1.49 (95% CI 1.24–1.79) for AF incidence using the most conservative adjustments provided in each individual study. After adjusting for three common risk factors (hypertension/blood pressure, BMI and various cardiovascular conditions) in addition to age, gender and possible others, the estimated risk for AF is 1.23 (95% CI 1.03–1.46). Our results are consistent with others, e.g., Dublin et al. estimated the RR for AF is 1.40 (95% CI 1.15–1.71) after adjusting for confounders including hypertension and BMI (Alves-Cabratosa et al., 2016) and the previous 2010 meta-analysis by Huxley et al. (2011) reported that patients with DM had a multivariate-adjusted RR 1.24 (95% CI 1.06–1.44) of new-onset AF than individuals without DM.

The association among DM, obesity and AF is complex, and reported results vary. For example, obesity was demonstrated to be more significantly associated with new-onset AF in Spanish hypertensive patients compared with DM (RR 1.41, 95% CI 1.22–1.64 vs. RR 1.11, 95% CI 1.06–1.16) (Alves-Cabratosa et al., 2016). Interestingly enough, another recent study indicated that DM is an independent risk factor for AF, but not hypertension and obesity in 11,956 subjects from rural Chinese areas (Sun et al., 2015).

### Trends of AF incidence in patients with DM

AF subtype was not analyzed in most of the studies. Only one study reported an association between cumulative exposure to DM and new-onset AF with all three AF subtypes (Dublin et al., 2010) and 6 studies reported the association between DM and one AF subtype. For the first time, our study has indicated that the risk estimate is not significantly different for any AF subtype. Since few studies have specifically addressed AF subtype and DM, future studies are warranted.

Patients with type 2 DM were exclusively enrolled in 7 out of the 29 selected studies, and there was only one study on the risk estimate of AF in patients with type 1 DM. Twenty-one studies did not explicitly define DM subtypes in their studies. Our study found no significant risk difference among these sub-groups. Interestingly enough, the study on type 1 DM by Dahlqvist et al. (2017) reported a similar gender difference in AF risk. Furthermore, they also observed the tendency of higher AF risk in younger people with DM.

AF incidence and prevalence are lower in women than in men. Sex hormones and delayed onset of cardiovascular disease in women are likely to contribute to these differences. However, the absolute number of women with AF exceeds that of men because women live longer (Perez et al., 2013; Gillis, 2017). Furthermore, women with AF are more likely to develop stroke than men with AF (Dublin et al., 2010; Sun et al., 2015). Our analysis confirms the increased risk of AF in women with DM compared to men, even though the average age between the two (54.16 ± 11.79 vs. 54.88 ± 12.87 year old) is the same. After adjusting for multiple comorbidities, the risk estimates are reduced but the gender difference for AF risk have become more pronounced (an increase from 17 to 24%).

The prevalence of AF in the general population is projected to more than double in the next few decades, becoming a global epidemic (Krijthe et al., 2013). On the other hand, DM is a common chronic disease and an increasing public health problem worldwide (Schnabel et al., 2015). Our study indicates that the trend of AF incidence in patients with DM is also increasing over time. The RR estimated for studies from 2001 to 2016 is significantly higher than the risk estimate for an equivalent number of studies from 1982 to 2001 (Figure 7). This potentially explains the reason why our overall risk estimate is higher than the estimated risk in the previous 2010 meta-analysis by Huxley et al. (2011). The increased AF incidence in diabetic patients may reflect enhanced awareness of this association and increased screening for AF. The trend also likely reflects the growing epidemic of obesity particularly in the developed world and the associated risk of developing metabolic syndrome.

Nevertheless, there are some important findings that are yet to be confirmed, such as the impact of median follow-up and age from new-onset DM on AF risk estimate. Some individual studies have suggested that the first 4–5 years after new-onset DM is the most vulnerable period for developing AF (Aksnes et al., 2008; Dublin et al., 2010; Pallisgaard et al., 2016); while some studies reported that AF incidence in patients with DM is most pronounced in young patients (Pallisgaard et al., 2016; Dahlqvist et al., 2017). However, most individual studies and our meta-analysis could not provide strong evidence to confirm these findings.

### Clinical significance

The recent review paper by Lau and his colleagues has proposed a promising integrated care model that incorporates risk factor management, including DM, as the fourth pillar of AF care alone with rate control, rhythm control and anticoagulation therapy (Lau et al., 2017). The benefits of lifestyle and risk factor modifications in atrial remodeling, disease progression and recurrence were clearly demonstrated in their previous studies (Pathak et al., 2014). The enriched knowledge with regard to DM and AF generated from our study will provide additional evidence to support and define a comprehensive lifestyle and optimal risk factor management for AF as an upstream therapy, as well as for stroke and mortality prevention (Lau et al., 2017).

### Study limitations

Our study has several limitations. The utility of a promising machine learning approach for publication selection is demonstrated in this study for the first time. However, future development and validation is needed for this approach to achieve its full capacity. The studies included in this meta-analysis are heterogeneous and include differences in patient demographics and marked variation in follow-up duration, though it is substantially reduced in subgroup studies. Furthermore, not all studies were adjusted for the multiple risk factors known to influence AF incidence. In addition, the efficacy of glycemic control on AF risk has not been assessed. Finally, the AF risk estimate for patients with DM in this study only demonstrates the possible causal association between DM and AF, the exact underlying mechanism remains elusive due to the complex nature of concurrent diseases and the limitations of current population-based clinical studies.

## Conclusions

We have demonstrated the utility of machine learning for meta-analyses. Our study has indicated that the AF risk estimates in patients with DM may be underestimated and has reinforced the view that DM is an independent risk factor for AF even after adjusting for other known concurrent risk factors.

## Author contributions

ZX and JZ conceived and designed the experiments; ZX, TL, GT, MG, MS, AG, and JZ data curation; PG, BS, MS, AG, and JZ investigation; ZX, TL, GT, and MG data analysis; All authors draft, reviewing and editing of the manuscript.

### Conflict of interest statement

The authors declare that the research was conducted in the absence of any commercial or financial relationships that could be construed as a potential conflict of interest.
