# Supplementary material for: A Machine Learning Aided Systematic Review and Meta-Analysis of the Relative Risk of Atrial Fibrillation in Patients With Diabetes Mellitus
Source: Front Physiol. 2018 Jul 3;9:835. doi: 10.3389/fphys.2018.00835 (PMC6037848; doi:10.3389/fphys.2018.00835)
Supplement: Supplementary file 1 [file Data_Sheet_1.PDF]

Supplementary Material

Supplementary Methods

The *R* programming language was used throughout the entire course of the study.[1] This open source software provided cutting edge functionalities for data mining, texting processing, machine learning[1] and statistical analysis[2].

1.1. Feature extraction

The challenge with meta-analysis stems from the tedious process required to manually select publications with relevant information. The presented approach demonstrated the ability for the algorithm to automatically separate a database of publications into distinct categories. To maximize the effectiveness of the machine learning algorithm, feature extraction had to be performed on the raw text to transform the data into a more recognizable form for the algorithm.

Natural language processing was the main technique used for feature extraction as it allowed for the text to be simplified, making it easier to obtain the meaning for each publication. The matrix was converted into a corpus structure which categorized all the words and arranged them for easy manipulation. Various text conversions were then done to strip off meaningless features. This included converting the words to lowercase, removing numbers, punctuation, special characters, extra white spaces, and removing stop words such as “the”, “and” or “a”. Stemming was then performed to reduce a derived word to its word stem. This essentially decreased the vocabulary by converting words with similar meaning to the exact same word. An example would be converting the words “running” and “ran” to the word “run”.

Since the preferred data format for machine learning algorithms is numerical, a document term matrix was created. This contained a summary of the number of occurrences for every single unique word in the title and abstract of each publication. The matrix was then normalized by weighing the values according to the frequency of each word which decreased the bias towards more often occurring words. Euclidean distances were then calculated for the frequencies to quantify the difference between a word or a group of words from another.

1.2. K means clustering

Since the number of different categories was unknown, the problem was unsupervised. This means that it was entirely up to the algorithm to independently recognize the features. Each research paper, with the features extracted, was considered as one data point. The K means clustering algorithm was used. This algorithm was chosen for its consistency in identifying subtle features to differentiate the categories as seen from test runs on a mock dataset. The clustering algorithm is an optimization implementation. During initialization, the algorithm selects random starting points, also called centroids, to begin clustering. The data points that were closest to a centroid were grouped together, forming a cluster. New centroids were then found by calculating the midpoints of the new clusters. This process was repeated so during every iteration, the centroids move a slight distance. The objective function was to minimize the distance between the position of the current centroids and the position of the centroids in the previous iteration.

The K means clustering algorithm was then used iteratively. During each iteration, the cluster of the smallest size was found and removed from the data set. This renewed data set was then clustered again with the smallest cluster removed. This process was repeated until the data set reached a size of less than 250 data points. 250 was chosen as it was manageable for manual checking.

1.3. Supervised machine learning classification

To identify which cluster was the most relevant to the topic of interest, a supervised machine learning classification algorithm was used. Another PubMed search was done to provide the algorithm with correct and incorrect examples to aid its identification. The key words "diabetes" and "atrial fibrillation" were searched only in the titles for each publication. A similar process was performed to extract the titles and abstracts from the PubMed studies downloaded. This smaller dataset was then manually searched and labelled "relevant" or "irrelevant" depending on the contents of the title and abstract.

In this study, 139 articles thought most likely to be relevant were identified by searching for the keywords "diabetes"/"diabetic" **and** "atrial fibrillation" in the **title**. These were reviewed and assigned to the labelled training set (26 studies that met the selection criteria and 113 that did not) by two experts.

The classification algorithm chosen was the maximum entropy algorithm. This was based on the principle of maximum entropy. The method involves using guess parameters that fit the training data and selecting the ones that produced the largest entropy, or un-orderliness, in the data. A comparison was done between different classification algorithms such as: support vector machine, neural-network, random forest, decision tree, boosting, bagging, bayesian and lasso and elastic-net regularized generalized linear models. During a simulation of 100 repeated runs, the maximum entropy classification algorithm had a significantly higher average accuracy for text classification in comparison to other classification algorithms ($\mathbf{p} < 0.0001$).

The individual publications in the different subgroups obtained from clustering were classified using the manually selected dataset as the training data. Since the machine learning algorithm had a fair amount of uncertainty, the subgroups were checked for their similarity with the training dataset to find the subgroup with the highest percentage similarity. This subgroup would then be considered to contain the highest probability of relevant publications. A search was also done to find the locations of the relevant publications obtained from the manual search for the training data.

Supplementary references

1. R Core Team. R: A language and environment for statistical computing. 2013.
2. Viechtbauer W. Conducting meta-analyses in R with the metafor package. *J Stat Softw*. 2010;36(3):1-48.

Table S1: All 21 cohort/randomized studies utilized in the systematic review and meta-analysis.

First Author (Year)	*Date of Enrolment*	*Country*	*N (%F)*	*Mean age (years)*	*Mean FU (years)*	*Incident Case of AF (%)*	*IR (per 1000 person-years)*	*DM diagnosis*	*AF diagnosis*	*Covariates in model*
Kannel et al (1982)	1948-1952	USA	5,191 (55)	48.8	10.4 (M) 10.9 (F)	98 (1.9%)	17.1(F) 21.5(M)	NA	ECG	age
Krahn et al (1995)	1948-1992	Canada	3,983 (0)	31.0	44.0	299 (7.5%)	<0.5 (age<50) 9.7 (age>70)	NA	ECG	age
Ruigómez et al (2002)	1996-1996	UK	6035 (54)	61.7	NA	1,035 (17.1%)	1.7	NA	ECG	age, sex and CVD
Frost et al (2005)	1993-2001	Denmark	47,589 (53)	56.0	5.7	553 (1.2%)	1.2 (F) 2.9 (M)	MRs	MRs	age, BMI, height, smoking, alcohol, Hyp, SBP, IHD, CHF and VHD
Aksnes et al (2008)	1997-2004	USA /Germany /Italy	15,245 (58)	67.0	4.2	780 (5.1%)	NA	FBG/ diabetes medication	ECG	age, sex, BMI, SBP, DBP, heart rate and potassium level
Watanabe et al (2008)	1996-1998	Japan	28,449 (66)	59.2	4.5	265 (1%)	1.3 (F) 4.1 (M)	FBG	ECG	age and sex
Nichols et al (2009)	1999-2004	USA	34,744 (49)	58.4	7.2	NA	6.6 (nondiabetics) 9.1 (diabetics)	FBG/ MRs	NA	age, sex, race, smoking, SBP, IHD, VHD, Hyp and HF
Rosengren et al (2009)	1970-1973	Sweden	6903 (0)	51.5	34.3 (max)	1,253 (18.2%)	7.5 (nondiabetics), 7.1 (diabetics)	SR	ECG	age

Smith et al (2010)	1991-1996	Sweden	30,441 (40)	58.0	11.2	1,430 (4.7%)	6.3(M) 3.1(F)	MRs/ diabetes medication	ECG	age
Huxley et al (2012)	1990-1992	USA	13,025 (50)	57.0	14.5	1,311 (10.1%)	4.51 (nondiabetics) 9.02 (diabetics)	FSG/ diabetes medication/MRs	ECG / MRs	age, sex, race, CHD,FSG, smoking, HF, SBP, Hyp, BMI
Schoen et al (2012)	2003-2011	USA	34,720 (100)	52.9	16.4	1,079 (3.1%)	1.99 (nondiabetics) 3.97 (diabetics)	SR	ECG / MRs	age, sex, CVD, IHD, BMI and Hyp
Fontes et al (2012)	1991-1994 1998-2001	USA	3,023 (55)	59.0	10.0	279 (9.3%)	NA	FBG	ECG	age, sex, SBP, Hyp, HF and BMI
Thacker et al (2013)	2001-2004	USA	1,385 (49)	69.2	0.5 (at least)	285 (100)%	NA	MRs/ diabetes medication	ECG / MRs	age, sex, BMI, Hyp, SBP, DBP, CHD, VHD, HF and stroke
Perez et al (2013)	1994-1998	USA	81,892 (100)	63.4	9.8	8,252 (10.1%)	NA	SR	MRs	age, sex, race, PAD, Hyp, HF, CHD, BMI, smoking, alcohol and HLD
Johnson et al (2014)	1974-1992	Sweden	7,066 (14)	57	26.2	983 (13.9%)	NA	FBG	MRs	age, sex, height, BMI, SBP and smoking
Staszewsky et al (2015)	2000-2010	Italy	825,330 (49)	65.1	9.0	57,965 (7.0%)	7.4 (nondiabetics) 10.4 (diabetics)	MRs/ diabetes medication	ECG / MRs	age, sex, medications, Hyp, HF and PHA
Son et al	2002-	Korea	206,013	49.0	6	3517	2.87	diabetes	NA	age, sex, BMI,

(2015)	2010		(41.2)			(1.7%)		medication		Hyp, IHD, HF
Zethelius et al (2015)	2005-2012	Sweden	83,162 (42)	64.1	6.8	4141 (5%)	9.2	MRs	ECG	age, sex, cholesterol, smoking, BMI, education
Thijs et al (2016)	2009-2012	USA/ Canada/ Europe	221 (35.7)	61.6	3.0	42 (19%)	NA	NA	ICM	none
Pallisgaard et al (2016)	1996-2012	Denmark	5,081,087 (51)	36.4	NA	NA	2.34 (age 18-39), 1.52 (age 40-46), 1.2 (age 65-74), 0.99 (age 75-100)	glucose-lowering medication	MRs	age, sex and year, Hpy, IHD, HF, VHD
Alves-Cabratosa et al (2016)	2006-2011	Spain	262,892 (42)	67.0	4.1	11,879 (4.5%)	10.4 (nondiabetics) 13.3 (diabetics)	MRs/ diabetes medication	MRs	age, SBP, DBP and LDLC

AF, atrial fibrillation; DM, diabetes mellitus; FU, follow-up; F, female; M, male; ECG,, electrocardiogram; FBG, fasting blood glucose; SR, self-reported; MRs, medical records; HF, heart failure; BMI, body mass index; CVD, cardiovascular disease; Hyp, hypertension; MI, myocardial infarction; CHF, congestive heart failure; VHD, valvular heart disease; SBP, systolic blood pressure; IHD, ischaemic heart disease; DBP, diastolic blood pressure; CHD, coronary heart disease; FSG, fasting serum glucose; PAD, peripheral arterial disease; HLD, Hyperlipidaemia; year, calendar year of patient data/time of study; PHA, past hospital admissions; LDLC, low-density lipoprotein cholesterol; IR, incidence rate; ICM, insertable cardiac monitoring.

Table S2: All 8 case-control studies utilized in the systematic review and meta-analysis.

First Author (Year)	*Date of Enrolment*	*Country*	*N (%F)*	*Mean Age (years)*	*Subjects*	*Prevalent AF*	*DM Diagnosis*	*AF Diagnosis*	*Covariates in model*
Kannel et al (1998)	1968-1998	USA	4,731 (56)	66.5	4,731 No Control Group	562 cases	MRs	ECG/ MRs	Age, sex, Hyp, CHD, HF, VHD
Alvarez et al (1999)	1996-1997	Spain	1,000 (NA)	64.6	300 AF, 700 controls	300 cases	NA	NA	none
Movahed et al (2005)	1990-2000	USA	845,748 (3)	65.1	293,124 DM, 552,624 controls	43,674 cases (14.9%), 57,077 controls (10.3%)	MRs	MRs	Age, sex, CHF, CAD and LVH
Johansen et al (2008)	2005	Norway	154 (31)	75.0	46 AF, 108 controls	NA	OGTT	ECG	None
Dublin et al (2010)	2001-2004	USA	3613 (59)	70.3	1,410 AF, 2,203 controls	NA	MRs	MRs	Age, sex, Hyp, BMI and year, race smoking, SBP, history, TC
Méndez-Bailón et al (2016)	2004-2013	Spain	214,457 (52)	72.7	214,457 AF, Unknown controls	NA	MRs	MRs	Age and year
Sun et al (2016)	2013-2013	China	11,341 (54)	53.8	1,171 DM, 10,170 controls	53 cases (4.7%), 14 controls (0.1%)	FBG	ECG	Age, sex, BMI, SBP, DBP, TC, TG, LDLC, HDLC, smoking, alcohol, MI, LLVEF and LVH
Dahlqvist et al (2017)	2001-2013	Sweden	216,238 (45)	35.5	36,258 DM, 179,980 controls	3631 cases (2%), 2882 controls	diabetes medication	MRs	age, sex, education, birthplace, CHD, HF, VHD, stroke, cancer

CAD, coronary artery disease; LVH, left ventricular hypertrophy; TC, total cholesterol; TG, triglycerides; HDLC, high-density lipoprotein cholesterol; LLVEF, low left ventricular ejection fraction; OGTT, oral glucose tolerance test; history, family history of AF; Other abbreviations see S1 Table.

Table S3: The 9 initially selected relevant studies that were excluded from the systematic review and meta-analysis.

First Author (Year)	*Date of Study*	*Country*	*Subjects*	*Mean Age (years)*	*Mean FU (years)*	*Incident Case of AF (%)*	*Covariates in Model*	*Reasons to Exclude*
Benjamin et al (1994)	1948-1952	USA	5,209	67	40.0	NA	Age, Hyp, smoking, diabetes, MI, CHF and VHD	Duplicated dataset
Benjamin et al (1998)	1948-1952	USA	1863 (52)	75.2	40.0	NA	NA	No quantitative estimate for the risk ratio for AF in DM patients was provided
Sun et al (2010)	NA	NA	NA	NA	NA	NA	NA	Review paper thus no additional information
Huxley et al (2011)	NA	NA	NA	NA	NA	NA	NA	Meta-Analysis paper thus no additional information
Sun et al (2015)	2013	China	11341	53.8	NA	NA	Age, gender, BMI, BP, FBG, TC, TG, smoking, drinking, physical activity, hyp, MI, history, LLVEF, rate and year	Duplicated dataset
Fatemi et al (2014)	1999-2013	USA/ Canada	10,082	62.2	7.2	159 (1.58%)	age, weight, DBP, HR and HF	No quantitative estimate for the risk ratio for AF in DM patients was provided
Grundvold et al (2015)	1999-2009	Sweden	7,169	60.0	4.6	287 (4.0%)	age, sex, BMI, AP and SBP	No quantitative estimate for the risk ratio for AF in DM patients was provided
Lee et al (2016)	2002-2007	Korea	40,500	62.0	5.9	1,261 (3.1%)	age, sex, Hyp, DLM, HF, COPD, MI, stroke or TIA, ESRD, low income	No quantitative estimate for the risk ratio for AF in DM patients was provided, only risk ratio for comparing DR to DM was given
Méndez-Bailón et al (2017)	2004-2013	Spain	214,457 (52)	72.7	NA	NA	age and year	Duplicated dataset

AP, angina pectoris; DLM, dyslipidaemia; ESRD, end-stage renal disease; COPD, chronic obstructive pulmonary disease; TIA, transient ischemic attack; AF, atrial fibrillation; DM, diabetes mellitus; FU, follow-up; F, female; M, male; ECG,, electrocardiogram; FBG, fasting blood glucose; SR, self-reported; MRs, medical records; HF, heart failure; BMI, body mass index; CVD, cardiovascular disease; Hyp, hypertension; MI, myocardial infarction; CHF, congestive heart failure; VHD, valvular heart disease; SBP, systolic blood pressure; IHD, ischaemic heart disease; DBP, diastolic blood pressure; CHD, coronary heart disease; FSG, fasting serum glucose; PAD, peripheral arterial disease; HLD, Hyperlipidaemia; year, calendar year of patient data/time of study; PHA, past hospital admissions; LDLC, low-density lipoprotein cholesterol; IR, incidence rate.

Table S4: Newcastle-Ottawa quality assessment scale *(*NOS*)/modified Jadad score* for the 21 cohort/randomized studies included.

First Author (Year)	*Represen-tativeness of the exposed cohort*	*Selection of the non-exposed cohort*	*Ascertai-nment of exposure*	*Outcome of interest not present at start of study*	*Comparability*	*Assessment of outcome*	*Adequacy of duration of follow-up*	*Adequacy of complete-ness of follow-up*	*Total score (0-9)*
Kannel et al (1982)	1	1	1	1		1	1	1	7
Krahn et al (1995)	1		1	1		1	1	1	6
Ruigómez et al (2002)	1	1	1			1	1	1	6
Frost et al (2005)	1	1	1	1	1 (age)	1	1	1	8
Aksnes et al (2008)	1	1	1	1		1	1	1	7
Watanabe et al (2008)	1	1	1	1	2 (age, gender)	1	1	1	9
Nichols et al (2009)	1	1	1	1	2 (age, gender)	1	1	1	9
Rosengren et al (2009)	1	1	1	1		1	1	1	7
Smith et al (2010)	1	1	1	1	1 (age)	1	1	1	8
Huxley et al (2012)	1	1	1	1	2 (age, gender)	1	1	1	9
Schoen et al (2012)	1	1	1	1		1	1	1	7

Thacker et al (2012)	1	1	1			1	1	1	6
Fontes et al (2012)	1		1	1		1	1	1	6
Perez et al (2013)	1	1	1	1		1	1	1	7
Johnson et al (2014)	1	1	1	1	1 (age)	1	1	1	8
*Thijs et al (2015)**									5
Staszewsky et al (2015)	1	1	1	1	2 (age, gender)	1	1	1	9
Zethelius et al (2015)	1	1	1	1	1 (gender)	1	1	1	8
Pallisgaard et al (2016)	1	1	1	1		1	1	1	7
Alves-Cabratosa et al (2016)	1	1	1	1	2 (age, gender)	1	1	1	9
Son et al (2016)	1	1	1	1	1 (gender)	1	1	1	8

* Modified Jadad score for the included randomized trial: 2 (randomization), 2 (concealment of allocation), 0 (double blinding) and 1 (withdraws/dropouts).

Table S5: *Newcastle-Ottawa quality assessment scale (NOS)* for the 8 case-control studies included in this study

First Author (Year)	*Adequate definition of cases*	*Represent-ativeness of cases*	*Selection of controls*	*Definition of controls*	*Comparability*	*Ascertainment of exposure*	*Same method of ascertain-ment for subjects*	*Non-response rate*	*Total score (0-9)*
Kannel et al (1998)	1	1				1	1	1	5
Álvarez et al (1999)	1	1		1	1 (age)	1	1	1	7
Movahed et al (2005)	1	1	1	1	2 (age, gender)	1	1	1	9
Johansen et al (2008)	1	1	1	1	2 (age, gender)	1	1	1	9
Dublin et al (2010)	1	1	1	1		1	1	1	7
Méndez-Bailó n et al (2016)	1	1	1	1		1	1	1	7
Sun et al (2016)	1	1	1	1	1 (gender)	1	1	1	8
Dahlqvist et al (2017)	1	1	1	1		1	1	1	7

Figure S1

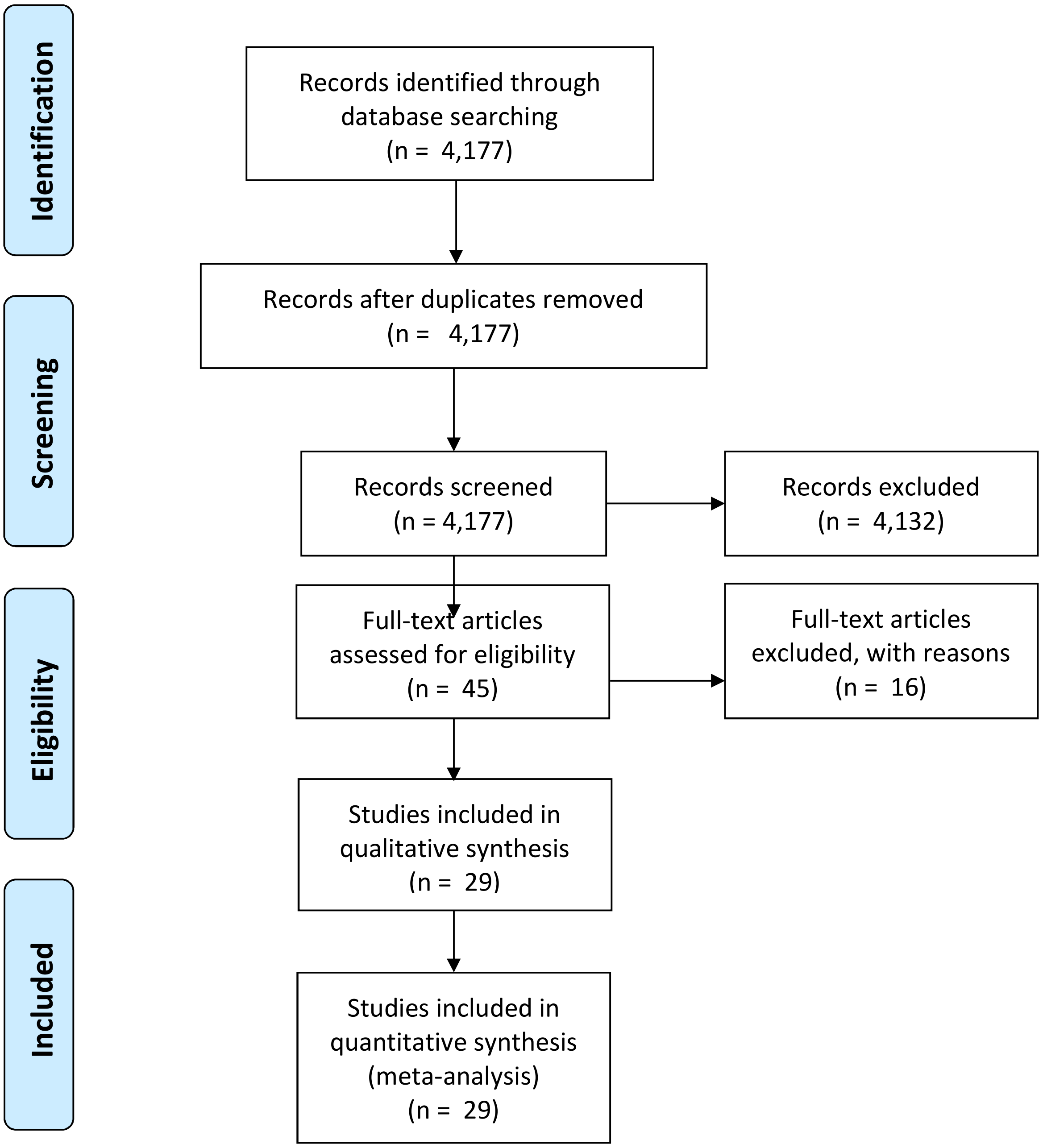

Figure S1. PRISMA 2009 Flow diagram.

Figure S2

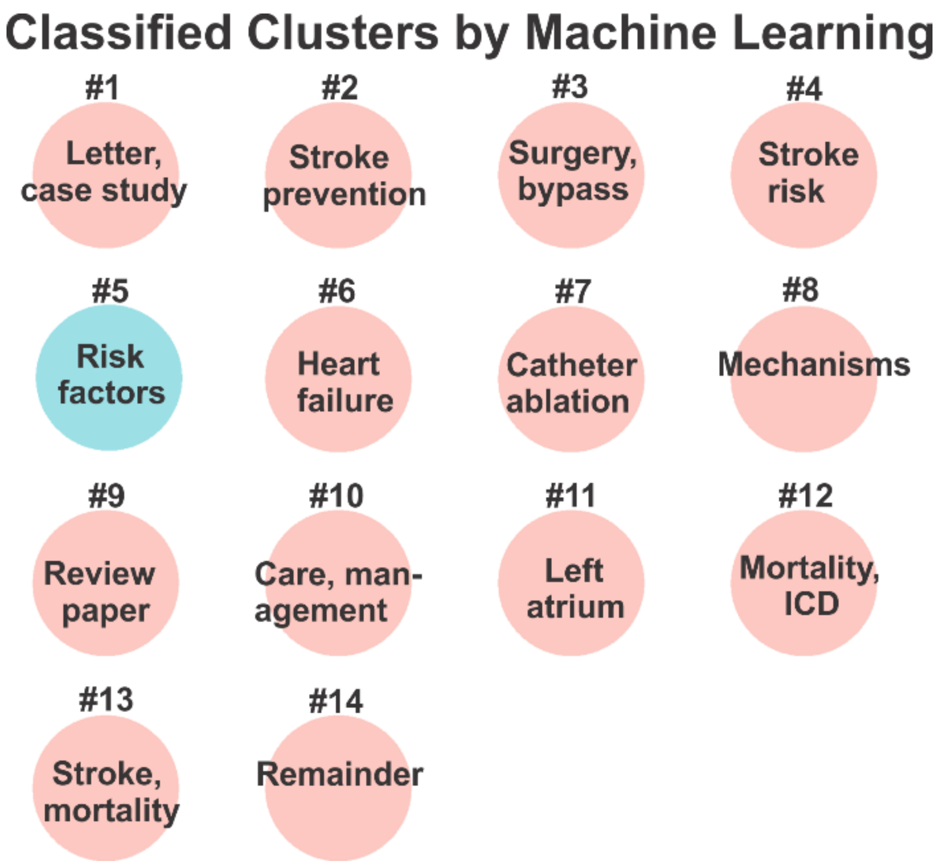

Figure S2. Visualization of the clusters obtained from K-means clustering and the associated key words of each cluster. Cluster #5 (with a key word of risk factor) was the cluster of interest and was successfully identified by the supervised machine learning classification algorithm. Cluster #14 includes all left over literature which were not a part of clusters #1-#13.

Figure S3

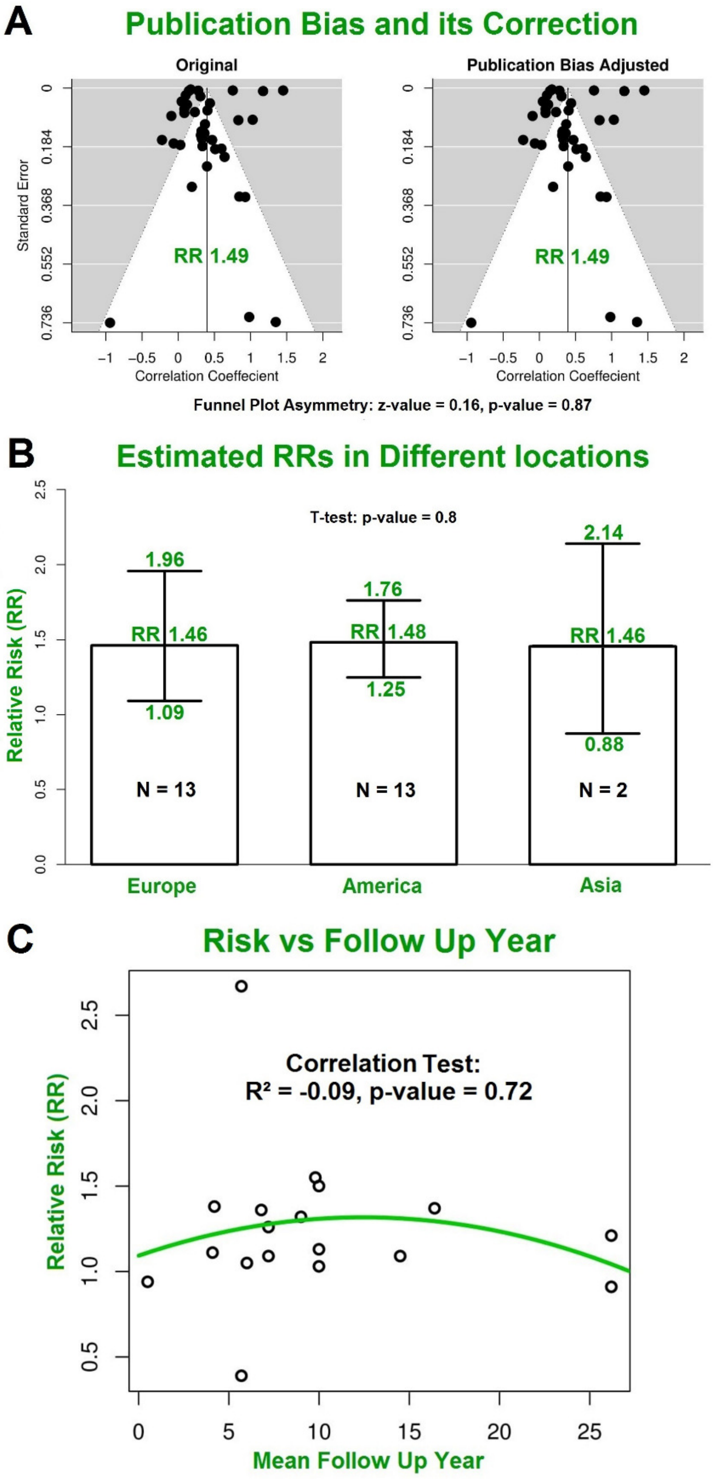

Figure S3. Publication bias, and the impact of study location and follow up years on estimated RRs. A. Funnel plot of the publication bias found no publication bias. **B.** Estimated RRs grouped by different continents. **C.** Individual RRs versus mean follow up year. RR, relative risk; CI, confidence interval.

Figure S4

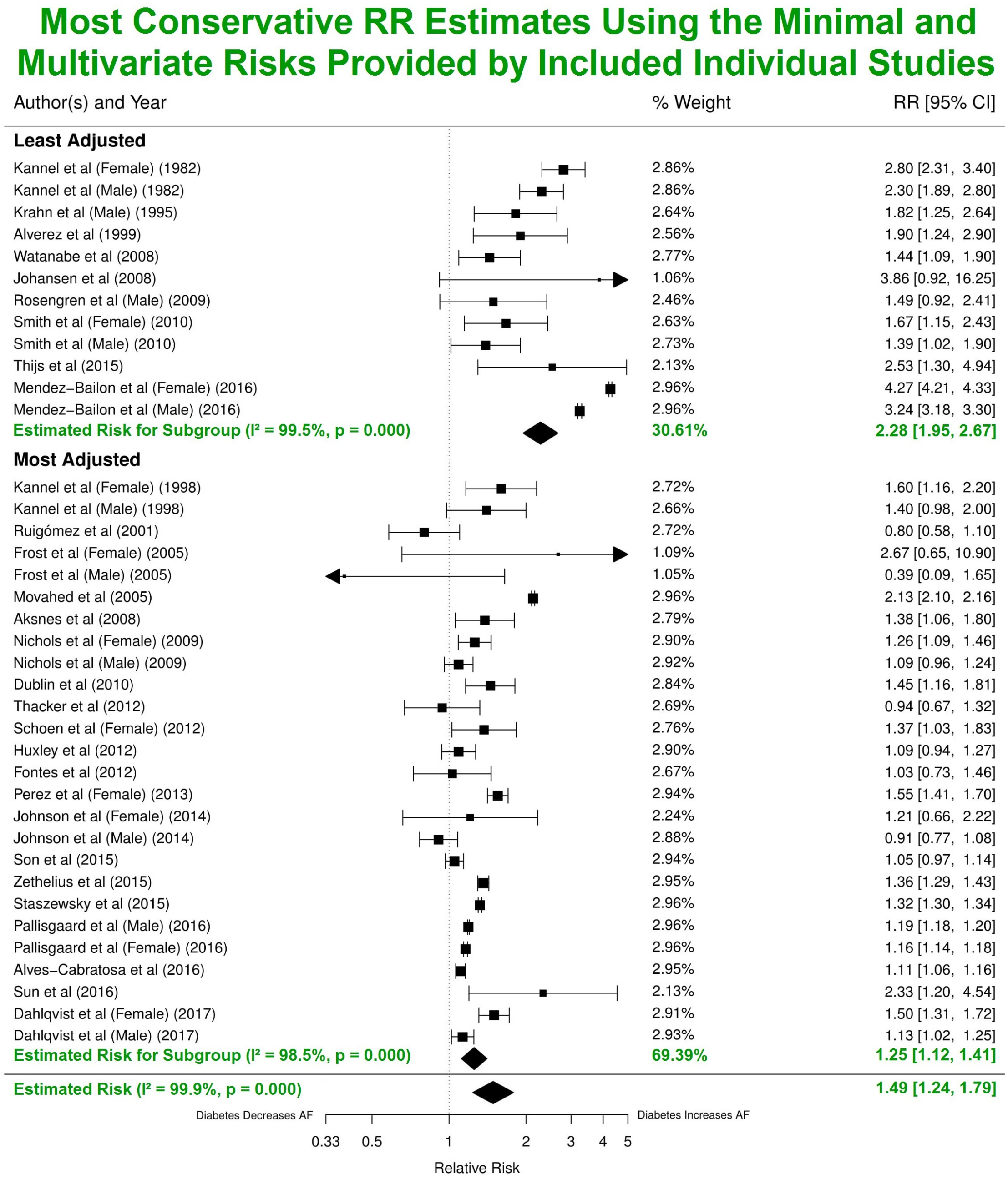

Figure S4. Estimated RRs of AF in patients with DM in reported minimal (age-and/or-gender/none) versus multivariate adjusted reports using the 29 studies. DM, diabetes milieus; AF, atrial fibrillation; RR, relative risk; CI, confidence interval.

Figure S5

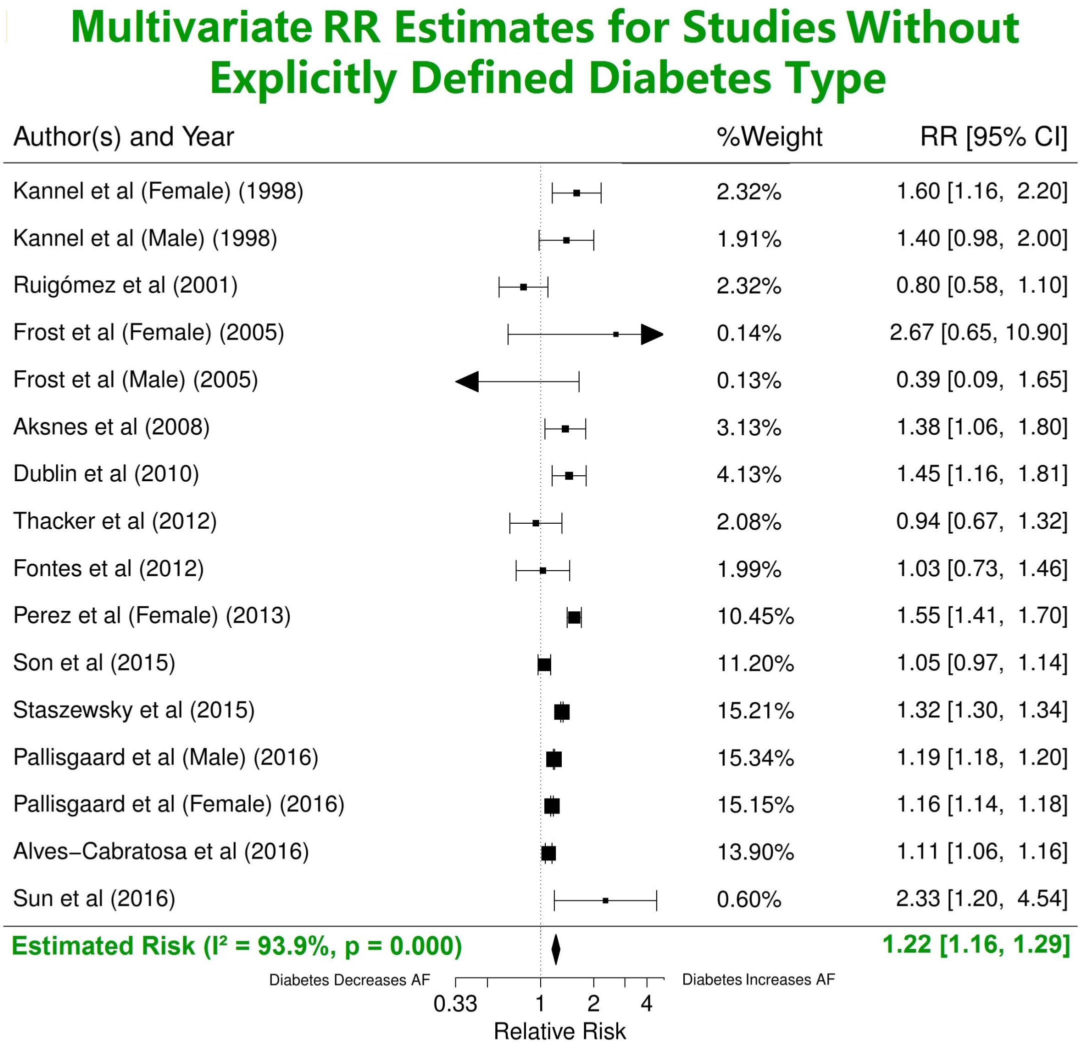

Figure S5. Estimated RRs of AF in patients without explicitly defined DM subtypes using the multivariate adjusted model. DM, diabetes milieus; AF, atrial fibrillation; RR, relative risk; CI, confidence interval.

Figure S6

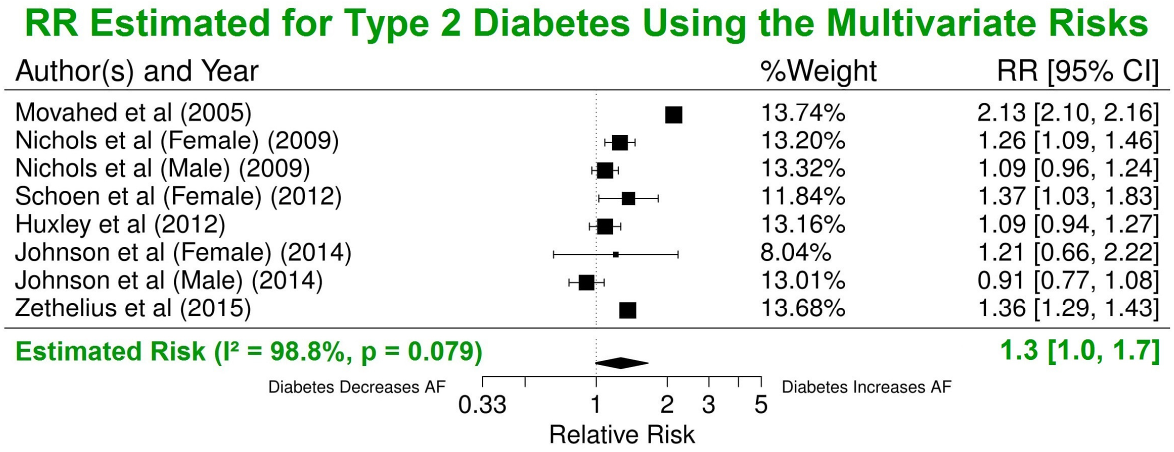

Figure S6. Estimated RRs of AF in patients with Type 2 DM using the multivariate adjusted model. DM, diabetes milieus; AF, atrial fibrillation; RR, relative risk; CI, confidence interval.
